# Prevalence of Human Papillomavirus Type-16 in Head and Neck Cancer Among the Chinese Population: A Meta-Analysis

**DOI:** 10.3389/fonc.2018.00619

**Published:** 2018-12-12

**Authors:** Lanwei Guo, Funa Yang, Yulin Yin, Shuzheng Liu, Peng Li, Xiaojun Zhang, Defeng Chen, Yang Liu, Jian Wang, Kai Wang, Yiming Zhu, Qing Lv, Xiaoyu Wang, Xibin Sun

**Affiliations:** ^1^Henan Office for Cancer Control and Research, The Affiliated Cancer Hospital of Zhengzhou University, Henan Cancer Hospital, Zhengzhou, China; ^2^Office of Cancer Screening, National Cancer Center/National Clinical Research Center for Cancer/Cancer Hospital, Chinese Academy of Medical Sciences and Peking Union Medical College, Beijing, China; ^3^Nursing Department, The Affiliated Cancer Hospital of Zhengzhou University, Henan Cancer Hospital, Zhengzhou, China; ^4^Department of Head and Neck Surgery, National Cancer Center/National Clinical Research Center for Cancer/Cancer Hospital, Chinese Academy of Medical Sciences and Peking Union Medical College, Beijing, China; ^5^Department of Head Neck and Thyroid Surgery, The Affiliated Cancer Hospital of Zhengzhou University, Henan Cancer Hospital, Zhengzhou, China

**Keywords:** head and neck cancer, human papillomavirus, prevalence, meta-analysis, China

## Abstract

**Background:** The burden of head and neck cancer in China is heavier, and studies have shown that it may be associated with HPV infection, especially high-risk HPV.

**Objectives:** We aimed to conduct a meta-analysis to estimate the high-risk HPV-16 prevalence of head and neck cancer in the Chinese population.

**Methods:** The reports on HPV and head and neck cancer in a Chinese population published between Jan 1, 2006 and Oct 23, 2018 were retrieved via WANFANG/CNKI/MEDLINE/EMBASE databases. The pooled prevalence and corresponding 95% confidence intervals was calculated by a random-effect model.

**Results:** The meta-analysis included a total of 2,896 head and neck cancer cases from 28 studies. Overall, the pooled HPV-16 prevalence among head and neck cancer cases was 24.7% (20.2–29.3%) in China, 31.6% (21.7–41.5%) in oropharyngeal cancer, 28.5% (18.2–38.7%) in laryngeal cancer and 14.9% (10.1–19.7%) in oral cancer, 25.3% (14.8–35.8%) in fresh or frozen biopsies and 25.0% (19.5–30.5%) in paraffin-embedded fixed biopsies, 36.5% (17.9–55.1%) by E6/E7 region and 14.3% (6.4–22.1%) by L1 region of HPV gene. The highest HPV-16 prevalence was found in Central China.

**Conclusions:** High prevalence of HPV-16 was found in the samples of Chinese head and neck cancers. Preventive HPV-vaccination may reduce the burden of HPV-related head and neck cancer in China.

## Introduction

As a member of the papillomavirus family of viruses, human papillomavirus (HPV) can infect humans by attacking the squamous cell of skin and mucous membranes, including those of the cervix, anogenital region and head and neck (1–3). As we all know, nasopharyngeal carcinoma is related to EB virus infection. Except that, more than 11% of the remaining squamous cell carcinomas of head and neck are caused by high-risk human papillomavirus infection (4), and the incidence of such subtypes is increasing year by year (5). However, the frequency of HPV infection in head and neck cancers varies between 3 and 84% in different studies (6). Based on the different nucleotide sequences, HPV can be divided into more than 200 genotypes by DNA sequencing, of which HPV16 and HPV18 are more closely related to malignant tumors as the main high-risk types (7, 8). However, unlike cervical and oral carcinogenesis, the role of HPV-16/18 in the development of other head and neck cancers has not been clearly defined.

Even in the same country, the HPV prevalence in head and neck cancers range widely (9). Demographic and racial factors, sample condition, cancer location and the viral detection method have been proposed to identify possible causes of differences in the results. However, as one of the most common subtype of HR-HPV, the HPV-16 prevalence in China has not been estimated so far.

The aim of the meta-analysis is to estimate the prevalence of HPV-16 detected in head and neck cancer cases and the different cancer sites, influence of regions, specimen types, and detection methods in China from all published studies of English and Chinese language literature.

## Materials and Methods

### Literature Search Strategies

This meta-analysis was reported following the guideline of Preferred Reporting Items for Systematic Reviews and Meta-Analyses (PRISMA) (10). The key words “human papillomavirus,” “papillomavirus infections,” “HPV,” “head and neck cancer,” “laryngeal cancer,” “oropharyngeal cancer,” “oral cancer,” “neck cancer,” “head cancer,” and corresponding carcinoma in English language or in Chinese language were used in combination to search. The retrieved databases included MEDLINE (via PubMed), Excerpta Medica database (EMBASE), Wanfang Data Knowledge Service Platform and Chinese National Knowledge Infrastructure (CNKI). Date of the literature was assigned between Jan 1, 2006 and Oct 23, 2018. Firstly, we excluded the duplicates. Secondly, we screened each title and abstract to evaluate its possible relevance. Thirdly, we downloaded full text for detailed evaluation if titles and abstracts weren't enough to make decision. All papers were independently reviewed by two authors (YFN and LSZ). Uncertainties and discrepancies were resolved by consensus after discussing with a senior researcher (SXB). If the data we needed were not explicitly reported or could not be derived from the papers, we also emailed authors to obtain related data. Additional studies were also identified using cross-referencing.

### Inclusion and Exclusion Criteria

According to the PRISMA statement, the literatures contained in this study must meets the following criteria: (a) to inform at least 10 cases of head and neck cancer confirmed by biopsy or histopathology, (b) to use polymerase chain reaction (PCR)-based methods (including type-specific PCR primers, broad-spectrum PCR primers, or a combination of both kinds of primers) or *in situ* hybridization (ISH) to amplify HPV DNA, (c) to report the prevalence of HPV-16 in head and neck cancer tissue samples with clear anatomical subsite.

The literatures excluded in this study were mainly due to the following reasons: were cellular or animal studies; were not conducted in Chinese; unable to extract or calculate the necessary data directly from the original article; without clear definition of anatomical subsite; reviews.

### Data Extraction

All studies included in the final meta-analysis extracted the following data: first author's name, publication year, geographical areas, cancer site, clinical stage, numbers of cases and HPV positive cases, HPV test method, specimen types (formalin-fixed paraffin-embedded biopsies [FFPE], fresh, or frozen biopsies [FF]).

### Statistical Analysis

Overall pooled point estimate and 95% confidence interval (95% *CI*) for HPV-16 prevalence were calculated through the method of DerSimonian and Laird (11) using the assumptions of a random-effects model. For a variety of HPV infections (including HPV-16), the multiple HPV types were classified into different types and the HPV-16 type-specific prevalence represents types for cases with either single HPV-16 infection and multiple HPV-16 infection.

Cochrane *Q* test (*P* < 0.10 indicated a high level of statistical heterogeneity) and *I*^2^ (values of 25, 50, and 75% corresponding to low, moderate and high degrees of heterogeneity, respectively) was used to assess the heterogeneity between eligible studies (12). Subgroup analyses for HPV-16 prevalence were subsequently carried out according to the geographical areas of the study origin, cancer site, year of publication, number of patients, HPV detection method and types of specimen. In the eligible studies, three studies, which contained different cancer sites, were treated as the separate studies. Meta-regression analyses were used to examine the association of the geographical areas of the study origin, cancer site, year of publication, number of patients, HPV detection method and types of specimen with the prevalence of HPV-16. Sensitivity analysis was also performed to assess the impact of each individual study on the strength and stability of the meta-analytic results. Each time, one study in the meta-analysis was excluded to show that study's impact on the overall impact size. Funnel plot and Begg adjusted rank correlation test for funnel plot asymmetry were performed to test any existing publication bias.

Statistical analysis was performed using STATA SE version 15.1 (StataCorp LP, College Station, TX, USA) for Windows. *P* < 0.05 with two-tailed was considered statistically significant.

## Results

### Systematic Review and Study Characteristics

**Figure 1:**
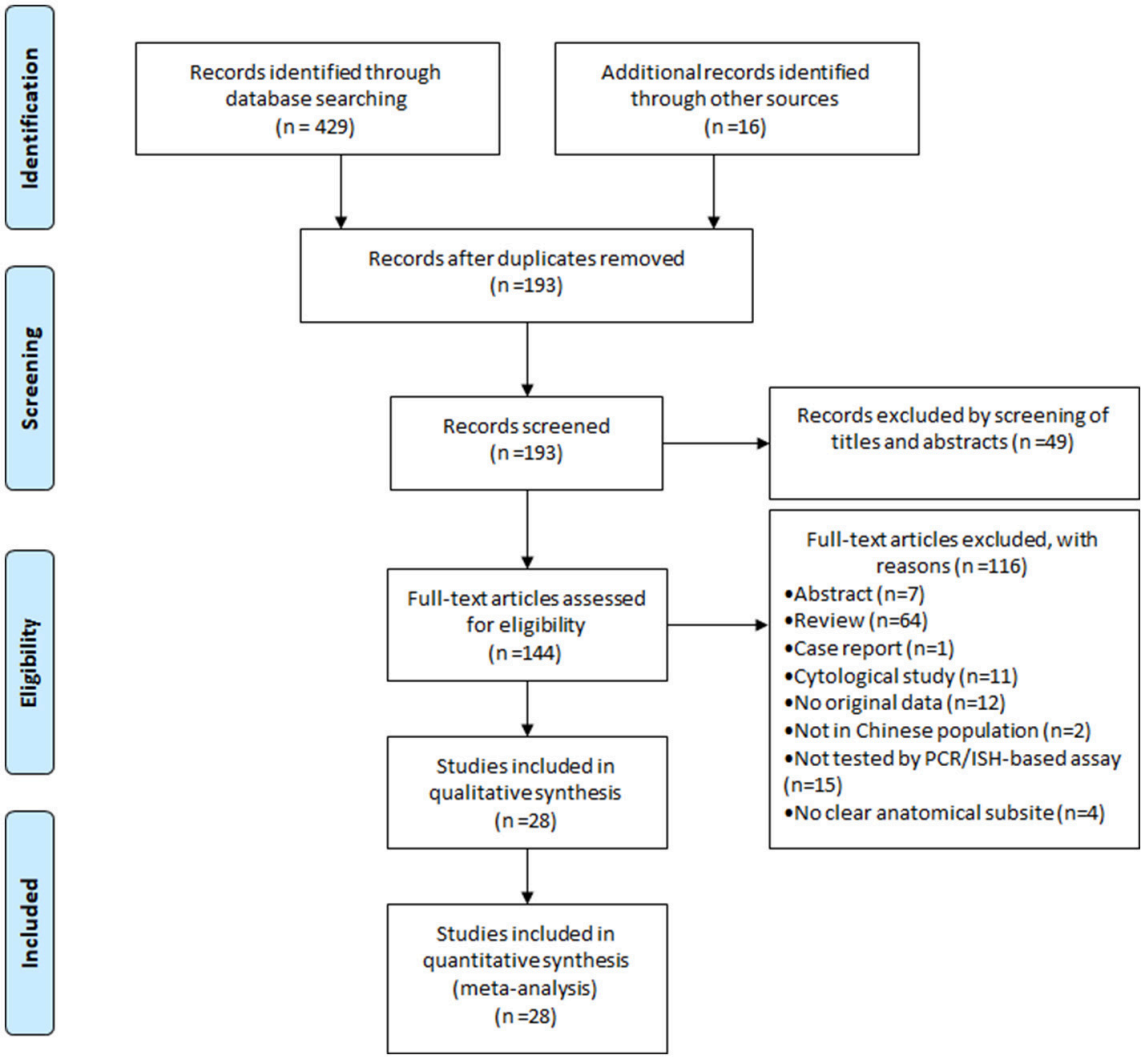
Flow diagram of systematic literature search on HPV-16 infection in head and neck cancer.

Figure 1 showed the flow diagram of systematic literature search. Generally speaking, the search strategy generated 445 citations, of which 193 were considered of potential value after screening of titles and abstracts and the full text was retrieved for detailed evaluation. 116 articles were subsequently excluded from the meta-analysis for various reasons, including 64 were reviews, 15 were not tested by PCR/ISH-based assay, 12 that did not provide *HR*s or *CI*s, 11 were cytological study, 7 were abstract, 4 had not clear anatomical subsite, 2 were not in Chinese population and 1 was case report. So, 28 studies (11 in English and 17 in Chinese) were eligible and included in this meta-analysis (13–40). Individual characteristics of the included 28 studies were summarized in Table 1. The size of the study samples ranged from 10 to 333 cases of head and neck cancer (median = 60). Summing up the studies, a total of 2,896 cases of head and neck cancer were identified. As shown in Table 1, more than half of the studies were conducted in Eastern China (*n* = 16, 57.14%), and the remaining studies distributed in three other regions of China as follows: 6 (21.43%) studies in Central China, 5 (17.86%) studies in Western China and 1 (3.57%) study in Northeastern China. 14 (50.00%) studies conducted in laryngeal cancer cases, 11 (39.29) conducted in oral cancer cases, and 8 (28.57%) conducted in oropharyngeal cancer cases. For HPV detection methods, 26 (92.86%) studies used PCR, and 2 (3.57%) studies used ISH. 21 (75.00%) studies used formalin-fixed paraffin-embedded biopsies (FFPE), and 7 (25.00%) studies used fresh or frozen biopsies. Besides, 7 (25.00%) studies used the gene detected from HPV E6/E7 region, and 5 (17.86%) used the gene detected from HPV L1 region.

**Table 1:** Studies included in the meta-analysis and their characteristics.

**References**	**Period of recruitment**	**Province**	**Region of China**	**Site**	**Stage**	**Method**	**Cases, *n***	**HPV-16 + ve, *n* (%)**	**Detection method**	**Specimen**
Wei and Qian (13)	NA	Guangxi	West	OC	NA	PCR	37	14 (37.8)	NA	FF
He (14)	NA	Hubei	Central	OC	NA	PCR	16	5 (31.3)	NA	FF
Peng et al. (15)	1998–2008	Chongqing	West	LC	I–III	PCR	123	67 (54.5)	E6/7	FFPE
Zhao et al. (16)	1999–2001	Hubei	Central	OC	I–IV	PCR	52	13 (25.0)	NA	FFPE
Liu et al. (17)	2000–2008	Beijing	East	LC	I–IV	PCR	84	23 (27.4)	L1	FFPE
Yao and Liu (18)	2005–2007	Shanxi	Central	LC	I–IV	PCR	30	22 (73.3)	E6	FFPE
Wang et al. (19)	2000–2008	Beijing	East	LC	I–IV	PCR	84	29 (34.5)	E6/7	FFPE
Cheng et al. (20)	2006–2009	Taiwan	East	OPC	III–IV	PCR	60	12 (20.0)	L1	FFPE
Huang et al. (21)	1999–2009	Beijing	East	OPC	I–IV	PCR	66	8 (12.1)	NA	FFPE
Lee et al. (22)	2004–2006	Taiwan	East	OC	III–IV	PCR	333	26 (7.8)	L1	FFPE
Lu et al. (23)	2011–2012	Shandong	East	LC	I–IV	PCR	57	2 (3.5)	NA	FF
Wu and Zhou (24)	2008–2011	Shanghai	East	LC	I–IV	PCR	46	2 (4.3)	E6/7	FFPE
Xue and Liu (25)	NA	Hubei	Central	OC	I–IV	PCR	30	8 (26.7)	NA	FFPE
Zhang et al. (26)	2004–2009	Beijing	East	OC, OPC, LC	NA	ISH	78	37 (47.4)	NA	FFPE
Gan et al. (27)	2009–2013	Hubei	Central	OC	I–IV	PCR	200	39 (19.5)	L1	FF
He et al. (28)	NA	Fujian	East	OC	I–IV	PCR	75	1 (1.3)	NA	FF
Wang et al. (29)	1999–2009	Guangdong	East	LC	I–II	PCR	163	3 (1.8)	L1	FFPE
Cui et al. (30)	2002–2011	Hunan	Central	OPC	I–IV	ISH	60	29 (48.3)	NA	FFPE
Guan et al. (31)	2009–2013	Shanghai	East	LC	NA	PCR	31	6 (19.4)	NA	FFPE
Chen et al. (32)	2012–2015	Fujian	East	OC	NA	PCR	178	6 (3.4)	NA	FFPE
Chor et al. (33)	2012–2014	Hongkong	East	OC, OPC, LC	NA	PCR	202	14 (6.9)	NA	FFPE
Fei et al. (34)	1995–2010	Yunnan	West	OPC	I–IV	PCR	60	20 (33.3)	E6	FFPE
Lam et al. (35)	2005–2009	Hongkong	East	OPC	I–IV	PCR	207	43 (20.8)	NA	FFPE
Lu et al. (36)	2010–2012	Guangdong	East	LC	I–IV	PCR	82	2 (2.4)	E2/6	FFPE
Ma et al. (37)	2012–2015	Sichuan	West	OC, OPC	I–IV	PCR	180	39 (21.7)	NA	FF
Wang et al. (38)	2014	Hainan	East	LC	NA	PCR	50	29 (58.0)	E6/7	FF
Zhang et al. (39)	2011–2016	Ningxia	West	LC	NA	PCR	101	9 (8.9)	NA	FFPE
Tong et al. (40)	NA	Heilongjiang	Northeast	LC	NA	PCR	211	132 (62.6)	NA	FFPE

*OC, oral cancer; LC, laryngeal cancer; OPC, oropharyngeal cancer; HPV-16 + ve, human papillomavirus 16 positive; PCR, polymerase chain reaction; ISH, in situ hybridization; FFPE, formalin-fixed paraffin-embedded biopsies; FF, fresh or frozen biopsies; NA, not available*.

### Meta-Analysis of HPV-16 Prevalence in Head and Neck Cancer Cases

**Figure 2:**
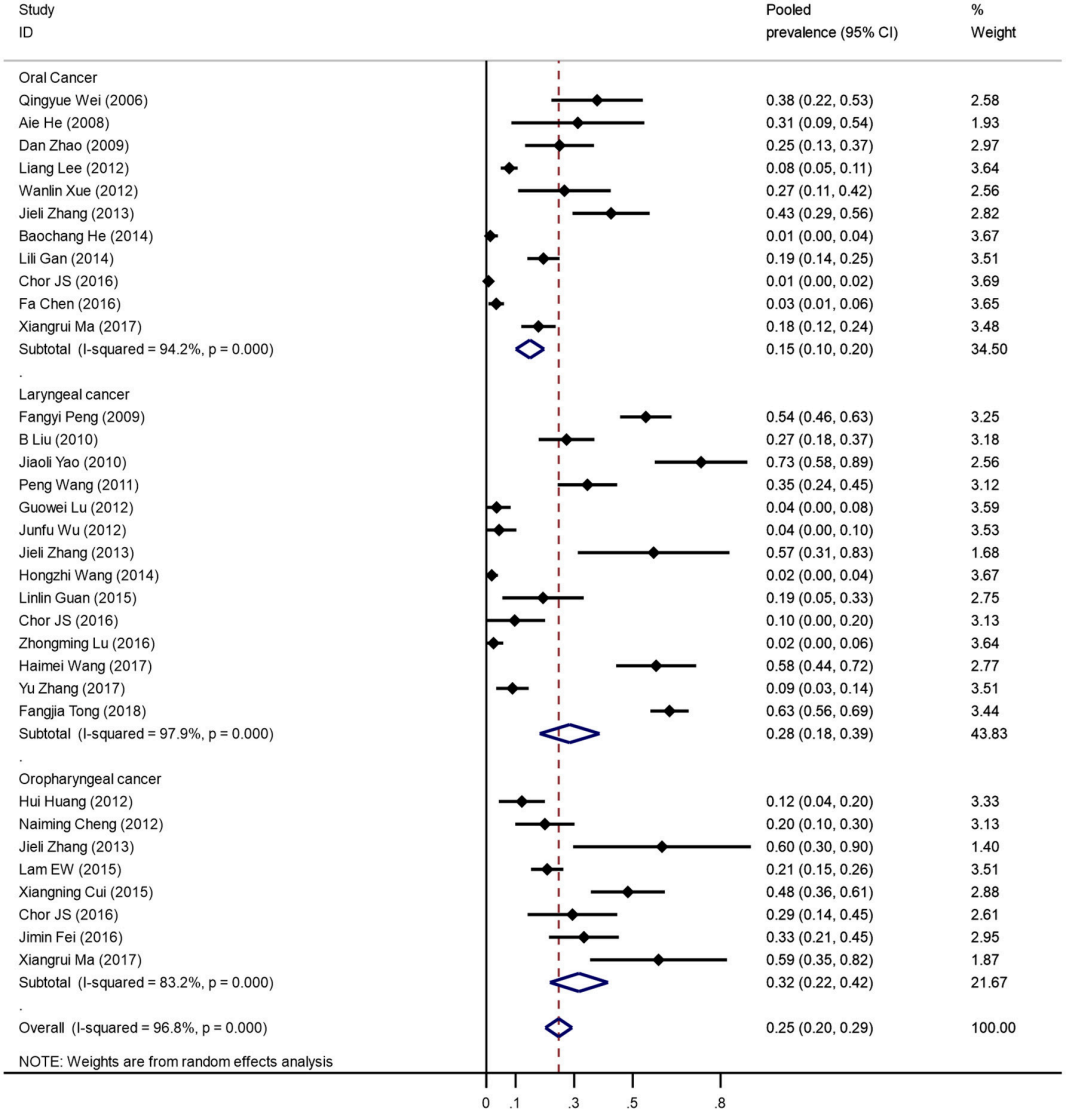
Forest plots of meta-analysis on HPV-16 prevalence in head and neck cancer tissue.

Figure 2 was the forest plot illustrated the individual and pooled prevalence estimates derived from a random effect model analysis. In this study, the prevalence of HPV-16 ranged from 1.3 to 58.0%. The pooled prevalence for HPV-16 was 24.7% (95% *CI*, 20.2–29.3%) in head and neck cancer cases in the Chinese population. Overall, high heterogeneity was observed in the studies included (*Q*-test *P*_heterogeneity_ < 0.001, *I*^2^ = 96.8%).

### Subgroup Analysis and Meta-Regression

Table 2 presents detailed results of subgroup analyses. As shown in Table 2, the highest pooled HPV-16 prevalence was observed in Central China (37.0%; 95% *CI*, 21.0–52.9%), followed by that in Western China (33.9%; 95% *CI*, 17.8–50.0%), and in Eastern China (14.5%; 95% *CI*, 10.9–18.1%). Oropharyngeal cancer had the highest infection rate of HPV-16 (31.6%; 95% *CI*, 21.7–41.5%), followed by laryngeal cancer (28.5%; 95% *CI*, 18.2–38.7%) and oral cancer (14.9%; 95% *CI*, 10.1–19.7%). Stratified analysis by published year showed that head and neck cancer before the year 2011 had the highest HPV-16 prevalence (41.6%; 95% *CI*, 26.7–56.4%) as compared with the period from 2011 to 2015 (18.9%; 95% *CI*, 13.7–24.1%) and the period from 2016 to 2017 (23.8%; 95% *CI*, 14.9–32.8%). Stratified analysis by number of patients showed that head and neck cancer patients < 100 has the higher HPV-16 prevalence (28.6%; 95% *CI*, 21.9–35.3%) as compared with those more than 100 (19.1%; 95% *CI*, 11.5–26.8%). HPV-16 prevalence with PCR method (22.1%; 95% *CI*, 17.6–26.7%) was lower than with ISH method (47.8%; 95% *CI*, 39.6–56.1%). Hierarchical analysis by specimen types showed that head and neck cancer in FF tissue (25.3%; 95% *CI*, 14.8–35.8%) had almost the same prevalence of HPV-16 compared with the FFPE tissue (25.0%; 95% *CI*, 19.5–30.5%). Regarding the HPV-16 test methods, the prevalence of head and neck cancer in the E6/E7 region of HPV gene (36.5%; 95% *CI*, 17.9–55.1%) was significantly higher than that in the L1 region (14.3%; 95% *CI*, 6.4–22.1%). In short, the estimated heterogeneity for studies included decreased to some extent but not disappeared. Meta-regression analyses found significant association of HPV-16 prevalence with HPV detection method (slope = 2.78, *P* = 0.027).

**Table 2:** Results of subgroup analyses for HPV-16 prevalence in head and neck cancer lesion.

**Variables**	**Studies, *n***	**Cases, *n***	**Prevalence (95% CI)**	**Heterogeneity test**	
				***p* for *Q* test**	***I*^**2**^ (%)**
Overall	33	2,896	24.7% (20.2–29.3%)	< 0.001	96.8
Region					
East	20	1,796	14.5% (10.9–18.1%)	< 0.001	93.6
Central	6	388	37.0% (21.0–52.9%)	< 0.001	90.2
West	6	501	33.9% (17.8–50.0%)	< 0.001	94.6
Site					
OC	11	1,275	14.9% (10.1–19.7%)	< 0.001	94.2
OPC	8	514	31.6% (21.7–41.5%)	< 0.001	83.2
LC	14	1,107	28.5% (18.2–38.7%)	< 0.001	97.9
Year					
2005–2010	6	342	41.6% (26.7–56.4%)	< 0.001	87.8
2011–2015	16	1,490	18.9% (13.7–24.1%)	< 0.001	93.8
2016–2017	11	1,064	23.8% (14.9–32.8%)	< 0.001	97.9
Number of patients					
< 100	23	1,080	28.6% (21.9–35.3%)	< 0.001	94.5
≥100	10	1,816	19.1% (11.5–26.8%)	< 0.001	98.4
Detection method					
PCR	29	2,758	22.1% (17.6–26.7%)	0.012	96.8
ISH	4	138	47.8% (39.6–56.1%)	0.631	0.0
Specimen					
FF	8	615	25.3% (14.8–35.8%)	< 0.001	95.6
FFPE	25	2,281	25.0% (19.5–30.5%)	< 0.001	97.1
Detection gene					
L1	5	840	14.3% (6.4–22.1%)	< 0.001	94.2
E6/E7	7	475	36.5% (17.9–55.1%)	< 0.001	97.8

*OC, oral cancer; LC, laryngeal cancer; OPC, oropharyngeal cancer; PCR, polymerase chain reaction; ISH, in situ hybridization; FFPE, formalin-fixed paraffin-embedded biopsies; FF, fresh or frozen biopsies; NA, not available*.

### Influence Analysis of Individual Studies

**Figure 3:**
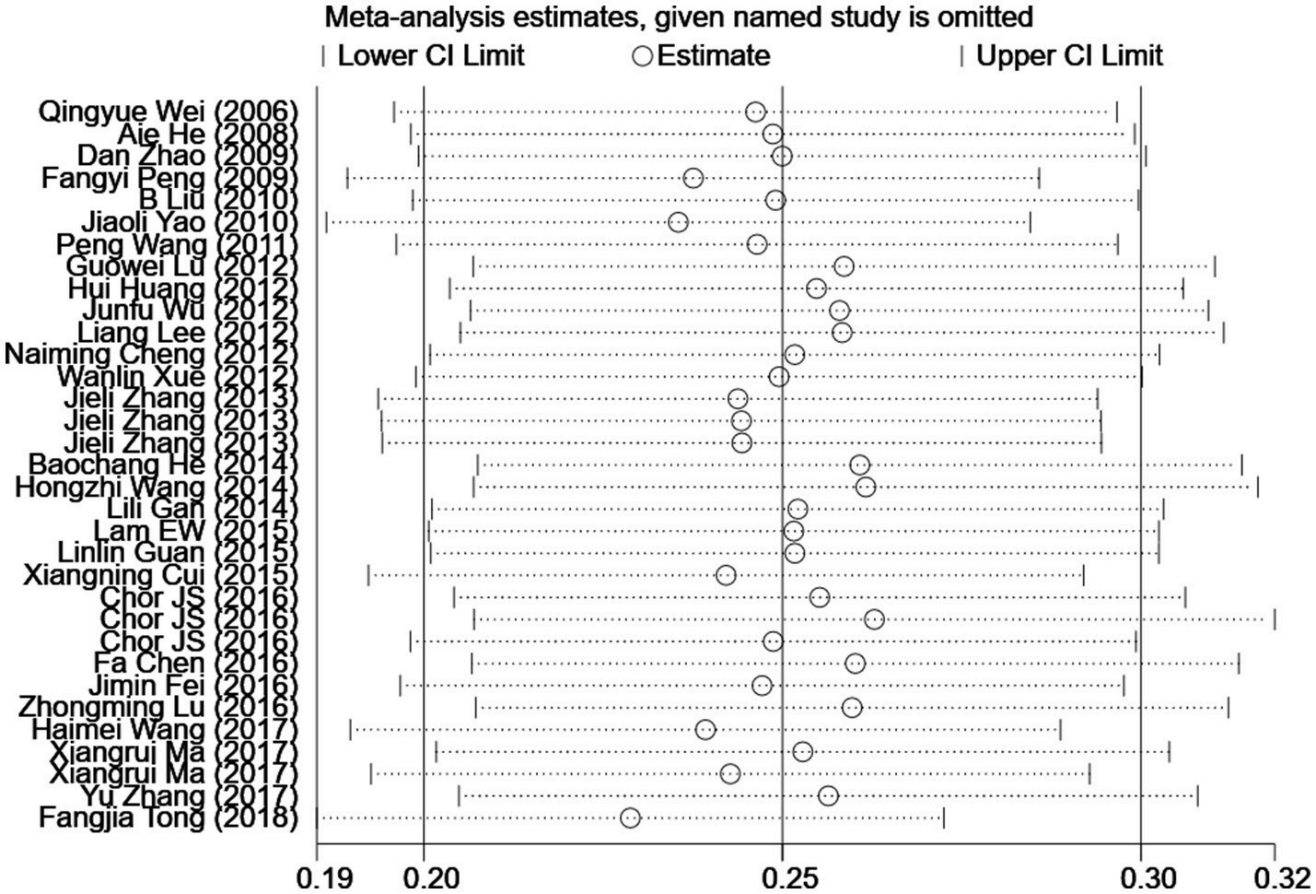
Influence analyses for individual studies on the summary effect.

To address the potential bias due to the quality of the included studies, we performed the sensitivity analysis by calculating pooled HPV-16 prevalence again when omitting one study at a time. Figure 3 showed the results of sensitivity analysis. The HPV-16 prevalence ranged from 23.0% (95% *CI*, 18.7–27.3%) to 26.4% (95% *CI*, 20.9–31.9%). Point estimates for all results of influence analysis were within 95% *CI*s of the pooled prevalence. The meta-analysis of pooled HPV-16 prevalence in head and neck cancer cases was not significantly affected by any of the 33 individual studies that was not analyzed, indicating that each study did not affect the stability of overall HPV-16 prevalence estimate.

### Publication Bias

**Figure 4:**
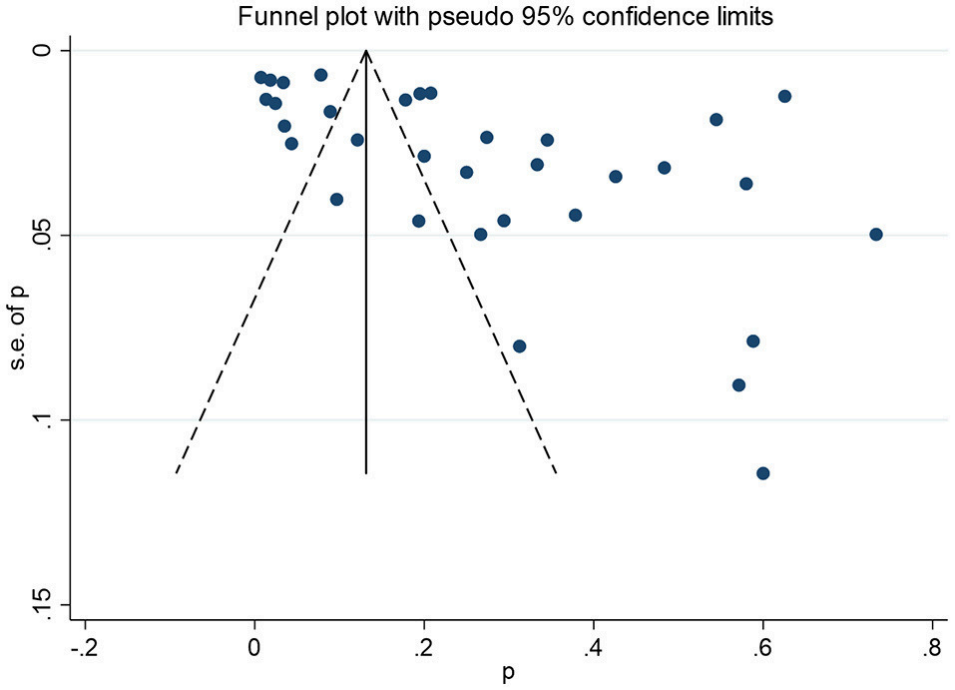
Funnel plots for publication bias.

The non-significant *P*-values of Begg's test (0.09), Eegg's test (0.07), and the near-symmetric funnel plot (Figure 4) demonstrated that there was no evidence of publication bias.

## Discussion

China has the highest burden of head and neck cancer around the world, which was rational for studying the prevalence of certain types of HPV (type-16) in China for the highly lethal cancer. As we know, this is the first meta-analysis to exploring the prevalence of HPV-16 in Chinese head and neck cancer tissues. The results of the meta-analysis showed that about 25% cases of head and neck cancer harbored HPV-16, indicating a high level of HPV 16 infection in head and neck cancer cases of China. Our results were little higher than the global prevalence (41).

Characterizing the HPV-16 prevalence in head and neck cancer is an important preliminary step in assessing the relationship between HPV-16 and head and neck cancer. Estimates of the HPV prevalence in various cancer sites of head and neck cancer vary considerably. Our meta-analysis showed that oropharyngeal cancer had the highest HPV-16 prevalence (31.6%), followed by that in laryngeal cancer (28.5%), and in oral cancer (14.9%). Interesting, the prevalence of HPV-16 in oropharyngeal cancer and oral cancer were much lower than Asian average, but much higher in laryngeal cancer (41). More cases involved and areas covered could be helpful in estimating the prevalence of HPV-16 in different head and neck cancer sites in the future.

The detection rate of HPV-16 DNA in FF tissue was found somewhat higher than that in FFPE tissue, given significant DNA degradation could be observed in FFPE tissue (42). The HPV infection status was determined on FFPE tissue in the majority of included studies. It is known that the low detection rate of HPV DNA occur with the fabric of FFPE, especially when a long DNA fragment was amplified.

Some possible reasons for variation in HPV prevalence among studies include small study sizes, different HPV testing, inter-laboratory variability, and manipulation of specimens leading to contamination (43–45). In our meta-analysis, PCR and ISH were just used as HPV detection methods, which focused on the HPV DNA detection method. Besides, for better PCR and ISH sensitivity and specificity, the literature time was limited from January 1, 2006 to Oct 23, 2018. HPV, a double-stranded circular DNA virus, encodes early proteins (E1, E2, E5, E6, and E7) and late proteins (L1, L2, and E4) with about 8,000 bp genome size (46, 47). When stratifying the L1 and E6/E7 gene fragments, we found that, in E6/E7 gene fragment (35.8%), the detection rate of HPV-16 DNA was much higher than in L1 gene fragment (14.3%). This is mainly due to the disruption of L1 region when HPV is integrated into the host genome (48), which may be an important event in promoting and triggering head and neck cancer.

The highlights of this meta-analysis includes a large sample size, both English and Chinese published studies are included with a strict inclusion criteria. By including English and Chinese studies, the selection bias caused by the publication language was avoided. Finally, by astricting studied published after 2006, limiting PCR and ISH detection methods and excluding studies without specific subsites, we tried to minimize the HPV prevalence variation as much as possible.

However, the meta-analysis has several limitations. First, the studies included in the meta-analysis are heterogeneous, which could be explained by changes in the population, the cancer site, the year of publication, the number of patients, the HPV detection method, the sample collection method, and the sensitivity of HPV primer PCR different protocols. To solve this issue, the random-effects model was used in the meta-analysis to combine data if significant heterogeneity was found. We directly tested heterogeneity by describing the HPV-16 prevalence in head and neck cancer cases by study area, cancer site, year of publication, number of patients, method of HPV detection and sample types. Of course, we have not been fully able to explain the heterogeneity. Even in stratified outcomes (for example, studies in different parts of China), the prevalence estimates are still uneven. Second, the study estimates may be biased because the accuracy of these estimates depends on the test method used and the type of HPV evaluated. That is, some studies use multiple probes or wide primers to detect multiple types of HPV, while other studies only detect HPV-16 type. Finally, the possibility of confounder cannot be ruled out. Limited studies have implicated the effect of age and smoking on the prevalence of HPV. We could not determine whether the variation of HPV prevalence was due to differences in environmental factors: age, sexual habits, smoking, alcohol consumption and other ethnic and cultural differences, as little or no information on these potential confounders in the studies involved. Therefore, studies with good designs to explore HPV infection by major confounding factors are likely to be required in future studies.

In short, the current meta-analysis provides a quantitative estimate of HPV-16 prevalence in head and neck cancer lesions in China. Although this review is a preliminary step in assessing the relationship between HPV-16 and head and neck cancer in China, it may be useful to evaluate the effect of HPV-16/18 prophylactic vaccines against carcinogenesis in the future. Considering that HPV infection plays an important role in the tumorigenesis of head and neck, other scientific studies deserve to be done in the future.

## Author Contributions

All authors have made substantial contributions to the conception and design of the study. LG and FY led protocol design, search, data extraction, statistical analysis, and manuscript drafting. YY and SL contributed to search, data extraction, and manuscript modifications. PL, XZ, DC, YL, JW, KW, YZ, QL, and XW contributed to quality assessment and revision of the manuscript. XS contributed to data interpretation and revision of the manuscript. All authors have reviewed and approved the final version.

### Conflict of Interest Statement

The authors declare that the research was conducted in the absence of any commercial or financial relationships that could be construed as a potential conflict of interest.
